# The desoxazoline asidiacyclamide analogue *cyclo*(Gly–Thr–D-Val–Thz–Ile–Thr–D-Val–Thz) acetonitrile monosolvate

**DOI:** 10.1107/S1600536811051543

**Published:** 2011-12-10

**Authors:** Akiko Asano, Mitsunobu Doi

**Affiliations:** a Osaka University of Pharmaceutical Sciences, Nasahara, Osaka 569-1094, Japan

## Abstract

The title peptide [systematic name: 4-(butan-2-yl)-7,20-bis­(1-hy­droxy­eth­yl)-10,23-bis­(propan-2-yl)-12,25-dithia-3,6,9,16,19,22,27,28-octa­aza­tricyclo­[22.2.1.1^11,14^]octa­cosa-1(26),11(28),13,24(27)-tetra­ene-2,5,8,15,18,21-hexone acetonitrile monosolvate], C_32_H_48_N_8_O_8_S_2_·CH_3_CN, an analogue of ascidiacyclamide (ASC) [*cyclo*(–Ile–Oxz–D-Val–Thz–)_2_], lies about a twofold rotation axis, so that the glycine (Gly) and isoleucine (Ile) residues are each disordered over two sites with equal occupancies. The acetonitrile mol­ecule is also located on a twofold axis passing through the C and N atoms. In the peptide, the thia­zole rings are faced to each other with a dihedral angle of 9.63 (15)° and intra­molecular N—H⋯O and O—H⋯O hydrogen bonds are observed. A bifurcated N—H⋯(O,O) hydrogen bond links the peptide mol­ecules into a layer parallel to the *ab* plane.

## Related literature

For general background to ascidiacyclamide, see: Hamamoto *et al.* (1983 ▶); Shioiri *et al.* (1987 ▶); Ishida *et al.* (1988 ▶); Degnan *et al.* (1989 ▶); Doi *et al.* (1999 ▶); Haberhauer & Rominger (2003 ▶). For related structures, see: Schmitz *et al.* (1989 ▶); Asano, Doi *et al.* (2001 ▶); Asano, Taniguchi *et al.* (2001 ▶); Asano *et al.* (2002 ▶, 2003 ▶, 2005 ▶).
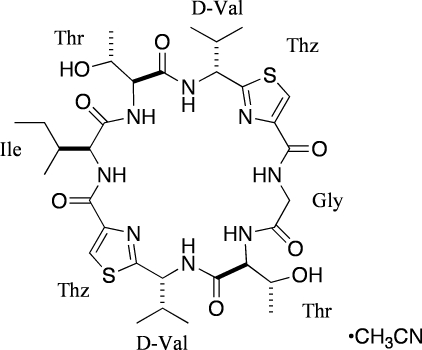


## Experimental

### 

#### Crystal data

C_32_H_48_N_8_O_8_S_2_·C_2_H_3_N
*M* *_r_* = 777.96
Orthorhombic, 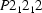

*a* = 18.2019 (9) Å
*b* = 10.4667 (5) Å
*c* = 11.0695 (6) Å
*V* = 2108.89 (18) Å^3^
*Z* = 2
Mo *K*α radiation
μ = 0.18 mm^−1^
*T* = 200 K
0.40 × 0.40 × 0.10 mm

#### Data collection

Bruker SMART APEX CCD area-detector diffractometer
Absorption correction: multi-scan (*SADABS*; Sheldrick, 1996 ▶) *T*_min_ = 0.909, *T*_max_ = 0.982
24275 measured reflections
4669 independent reflections
4359 reflections with *I* > 2σ(*I*)
*R*_int_ = 0.026

#### Refinement

*R*[*F* ^2^ > 2σ(*F* ^2^)] = 0.075
*wR*(*F* ^2^) = 0.213
*S* = 1.09
4669 reflections
269 parameters
H-atom parameters constrained
Δρ_max_ = 0.92 e Å^−3^
Δρ_min_ = −1.42 e Å^−3^
Absolute structure: Flack (1983 ▶), 2016 Friedel pairs
Flack parameter: 0.11 (13)

### 

Data collection: *SMART* (Bruker, 1998 ▶); cell refinement: *SAINT-Plus* (Bruker, 1998 ▶); data reduction: *SAINT-Plus*; program(s) used to solve structure: *SHELXS97* (Sheldrick, 2008 ▶); program(s) used to refine structure: *SHELXL97* (Sheldrick, 2008 ▶); molecular graphics: *PLATON* (Spek, 2009 ▶); software used to prepare material for publication: *SHELXL97*.

## Supplementary Material

Crystal structure: contains datablock(s) global, I. DOI: 10.1107/S1600536811051543/is5015sup1.cif

Structure factors: contains datablock(s) I. DOI: 10.1107/S1600536811051543/is5015Isup2.hkl

Supplementary material file. DOI: 10.1107/S1600536811051543/is5015Isup3.mol

Additional supplementary materials:  crystallographic information; 3D view; checkCIF report

## Figures and Tables

**Table 1:** Hydrogen-bond geometry (Å, °)

*D*—H⋯*A*	*D*—H	H⋯*A*	*D*⋯*A*	*D*—H⋯*A*
O22—H22⋯O16	0.84	1.87	2.674 (4)	160
N11—H11⋯O16^i^	0.88	2.12	2.984 (3)	166
N21—H21⋯O22^ii^	0.88	2.29	2.953 (4)	132
N21—H21⋯O24^ii^	0.88	2.25	3.013 (4)	145
N31—H31⋯O44^i^	0.88	2.27	3.074 (3)	152

Symmetry codes: (i) 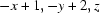; (ii) 
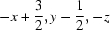
.

**Fig. 1.**
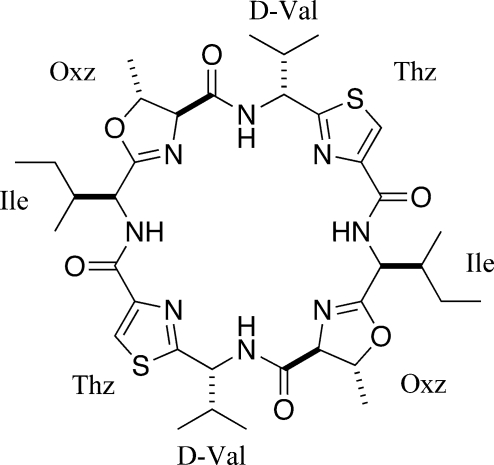
Chemical structure of ascidiacyclamide.

**Fig. 2.**
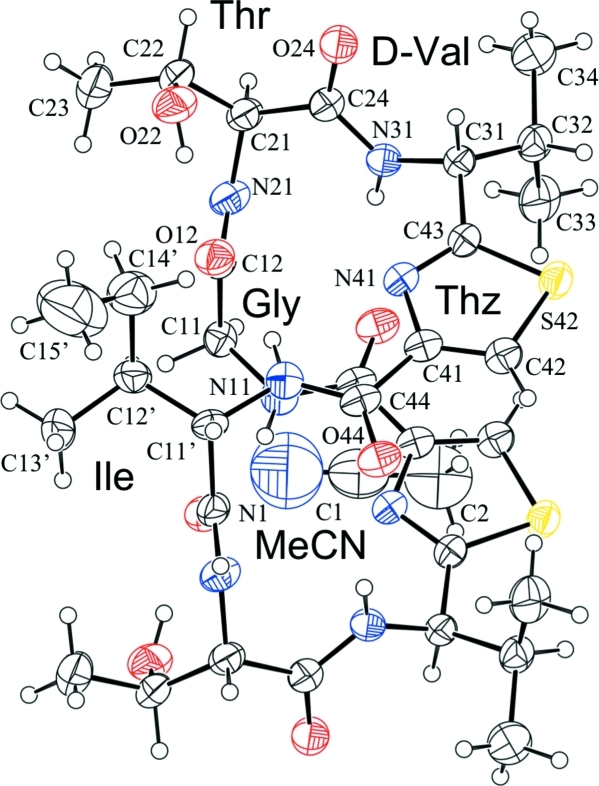
The structure of the title compound, with displacement ellipsoids drawn at the 30% probability level and H atoms with arbitrary radius. The molecule is located on the twofold axis. The crystallographically duplicated atoms are drawn in figure to show the whole peptide structure.

## supplementary crystallographic information

### Comment

Ascidiacyclamide (ASC) is an unique cyclic peptide accommodating the unusual amino acids of oxazoline (Oxz) and thiazole (Thz) units (Fig. 1) (Hamamoto *et al.*, 1983; Shioiri *et al.*, 1987). These five-membered rings limit the rotations of N—Cα bonds (the φ angel) causing the conformational restrictions to the molecule. The title peptide has only the Thz units and the conformation is more flexible than ASC. The first residue, Ile, is replaced with Gly which has less steric hindrances. This analogue is designed for the fundamental studies to control the peptide structure and to develop new class analogues of ASC.

The structure is shown in Fig. 2. The peptide and MeCN molecules are located on the two-fold axis and the figure is drawn to show the whole molecular structure with the crystallographically independent atoms and duplicated atoms related by the symmetry of (3/2 - *x*, *y* - 1/2, -*z*). The Gly and Ile residues are coexisted with the disordering state, but they are independently drawn in the figure for clarity. The peptide molecule is folded at the Thr residue and the Thz rings are faced to each other. The atomic radius of sulfur atom (S42), which is larger than that of carbon, causes a slight tilt between the Thz ring with a dihedral angle of 9.63 (15)°. The distance between the S42 atoms of Thz is 4.303 (1) Å. These structural characteristics indicate the similarity with the folded forms of ASC analogues (Schmitz *et al.*, 1989; Asano, Doi *et al.*, 2001).

The intramolecular hydrogen bonds are formed between the amide groups related by twofold axis: N11···O16 = 2.984 (3) and N31···O44 = 3.074 (3) Å (Table 1). These interactions stabilize the folded structure. The N21 atom of Thr is hydrogen-bonding to the O22 and O24 atoms of the adjacent molecule related by the symmetry of (3/2 - *x*, *y* - 1/2, -*z*).

The hydroxyl group of Thr (O22) is hydrogen-bonding to the preceding carbonyl group (O16) of Gly or Ile forming 7-membered ring. This interaction seems to be caused by 3*R*-configuration of Cβ atom (C22). In the previous studies for desoxazoline ASC analogues (Asano, Doi *et al.*, 2001), the hydroxyl group of *allo*-Thr (3S-configuration) is interacted with its carbonyl group (O24 in this structure) forming 6-membered ring. In this structure, the O22···O24 hydrogen bond causing the rotation of Cα—Cβ bond would result the steric hindrances at the methyl group (C23). Therefore, the O22···O16 hydrogen bond is formed at the Thr residue. The configuration of Cβ atom interestingly contributes the direction of hydroxyl group of *allo*-Thr and Thr.

### Experimental

The title peptide was synthesized by the previously described method (Asano *et al.*, 2005). The peptide was purified by using preparative thin-layer chromatography. Single crystals were grown from an aqueous MeCN solution.

### Refinement

The side chain atoms of Gly and Ile residues were observed as disordered state, and refined with the site of occupancy 0.5. C-bound H atoms were placed in idealized positions with C—H distances 0.95–1.00 Å and treated as riding, with *U*_iso_(H) = 1.2*U*_eq_(C) or 1.5*U*_eq_(methyl C). N-bound H atoms were also placed in idealized positions with N—H distances 0.88 Å and treated as riding, with *U*_iso_(H) = 1.2*U*_eq_(N). The H atom of hydroxyl group (O22) was placed guided by difference maps, based on hydrogen bonding considerations, and fixed during the refinement, with *U*_iso_(H) = 1.5*U*_eq_(O). The highest residual peak was located at (0.0200,0.7970,0.3512), 0.48 Å from atom H15C, and the deepest residual hole at (0, 0, 0.9130), 0.06 Å from atom N1.

### Crystal data

**Table:**

C_32_H_48_N_8_O_8_S_2_·C_2_H_3_N	*F*(000) = 828
*M**_r_* = 777.96	*D*_x_ = 1.225 Mg m^−^^3^
Orthorhombic, *P*2_1_2_1_2	Mo *K*α radiation, λ = 0.71073 Å
Hall symbol: P 2 2ab	Cell parameters from 8940 reflections
*a* = 18.2019 (9) Å	θ = 2.2–25.5°
*b* = 10.4667 (5) Å	µ = 0.18 mm^−^^1^
*c* = 11.0695 (6) Å	*T* = 200 K
*V* = 2108.89 (18) Å^3^	Plate, colourless
*Z* = 2	0.40 × 0.40 × 0.10 mm

### Data collection

**Table:**

Bruker SMART APEX CCD area-detector diffractometer	4669 independent reflections
Radiation source: MacScience, M18XCE rotating anode	4359 reflections with *I* > 2σ(*I*)
graphite	*R*_int_ = 0.026
Detector resolution: 8.366 pixels mm^-1^	θ_max_ = 27.1°, θ_min_ = 1.8°
ω–scan	*h* = −23→23
Absorption correction: multi-scan (*SADABS*; Sheldrick, 1996)	*k* = −13→13
*T*_min_ = 0.909, *T*_max_ = 0.982	*l* = −14→14
24275 measured reflections	

### Refinement

**Table:**

Refinement on *F*^2^	H-atom parameters constrained
Least-squares matrix: full	*w* = 1/[σ^2^(*F*_o_^2^) + (0.1468*P*)^2^ + 0.6849*P*] where *P* = (*F*_o_^2^ + 2*F*_c_^2^)/3
*R*[*F*^2^ > 2σ(*F*^2^)] = 0.075	(Δ/σ)_max_ = 0.017
*wR*(*F*^2^) = 0.213	Δρ_max_ = 0.92 e Å^−^^3^
*S* = 1.09	Δρ_min_ = −1.42 e Å^−^^3^
4669 reflections	Absolute structure: Flack (1983), 2016 Friedel pairs
269 parameters	Flack parameter: 0.11 (13)
0 restraints	

### Special details

**Table:**

Geometry. Thz-Thz plane angle = 9.54 °, S(Thz)···S(Thz) = 4.303 A (mercury)

### Fractional atomic coordinates and isotropic or equivalent isotropic displacement parameters (Å^2^)

**Table:**

	*x*	*y*	*z*	*U*_iso_*/*U*_eq_	Occ. (<1)
C1	0.5000	0.5000	0.2003 (14)	0.108 (3)	
C2	0.5000	0.5000	0.3182 (14)	0.130 (4)	
H2A	0.4497	0.5116	0.3477	0.195*	0.50
H2B	0.5309	0.5700	0.3477	0.195*	0.50
H2C	0.5194	0.4184	0.3477	0.195*	0.50
N1	0.5000	0.5000	0.0925 (9)	0.107 (3)	
N11	0.51634 (15)	0.8447 (3)	0.0697 (3)	0.0418 (6)	
H11	0.4732	0.8769	0.0505	0.050*	
C11	0.572 (3)	0.812 (4)	−0.024 (4)	0.045 (6)	0.50
H11A	0.5480	0.8033	−0.1035	0.054*	0.50
H11B	0.5956	0.7293	−0.0035	0.054*	0.50
C11'	0.564 (2)	0.827 (5)	−0.038 (4)	0.044 (6)	0.50
H11C	0.5836	0.7382	−0.0345	0.053*	0.50
C12'	0.5197 (4)	0.8380 (9)	−0.1579 (7)	0.0539 (18)	0.50
H12	0.4950	0.9233	−0.1568	0.065*	0.50
C13'	0.5723 (5)	0.8385 (12)	−0.2662 (7)	0.070 (2)	0.50
H13A	0.6090	0.9060	−0.2554	0.106*	0.50
H13B	0.5444	0.8543	−0.3404	0.106*	0.50
H13C	0.5970	0.7555	−0.2718	0.106*	0.50
C14'	0.4574 (6)	0.7354 (11)	−0.1663 (12)	0.081 (3)	0.50
H14A	0.4481	0.6952	−0.0868	0.097*	0.50
H14B	0.4111	0.7733	−0.1966	0.097*	0.50
C15'	0.4887 (8)	0.6390 (16)	−0.2562 (14)	0.116 (5)	0.50
H15A	0.4527	0.5710	−0.2701	0.174*	0.50
H15B	0.5339	0.6020	−0.2234	0.174*	0.50
H15C	0.4995	0.6822	−0.3327	0.174*	0.50
C16	0.62923 (19)	0.9158 (3)	−0.0285 (3)	0.0467 (8)	
O16	0.61582 (14)	1.0319 (3)	−0.0393 (2)	0.0525 (6)	
N21	0.69723 (16)	0.8697 (3)	−0.0161 (3)	0.0505 (7)	
H21	0.7022	0.7861	−0.0176	0.061*	
C21	0.76449 (19)	0.9454 (4)	0.0001 (3)	0.0482 (8)	
H21A	0.8056	0.8822	0.0049	0.058*	
C22	0.7838 (2)	1.0347 (4)	−0.1057 (3)	0.0564 (9)	
H22A	0.8356	1.0632	−0.0929	0.068*	
O22	0.73999 (17)	1.1466 (3)	−0.1100 (3)	0.0661 (8)	
H22	0.6964	1.1278	−0.0921	0.099*	
C23	0.7822 (3)	0.9632 (6)	−0.2260 (4)	0.0814 (15)	
H23A	0.7948	1.0222	−0.2916	0.122*	
H23B	0.7329	0.9285	−0.2397	0.122*	
H23C	0.8179	0.8932	−0.2240	0.122*	
C24	0.76604 (19)	1.0189 (4)	0.1201 (3)	0.0459 (7)	
O24	0.80873 (17)	1.1097 (3)	0.1328 (2)	0.0615 (7)	
N31	0.27760 (14)	1.0230 (3)	0.2096 (2)	0.0403 (6)	
H31	0.3012	1.0958	0.2006	0.048*	
C31	0.28693 (17)	0.9514 (3)	0.3208 (3)	0.0390 (6)	
H31A	0.2578	0.8710	0.3114	0.047*	
C32	0.2561 (2)	1.0205 (4)	0.4338 (3)	0.0504 (8)	
H32	0.2707	0.9700	0.5066	0.061*	
C33	0.2876 (3)	1.1554 (5)	0.4473 (4)	0.0688 (12)	
H33A	0.3413	1.1509	0.4503	0.103*	
H33B	0.2691	1.1939	0.5221	0.103*	
H33C	0.2724	1.2076	0.3782	0.103*	
C34	0.1722 (3)	1.0227 (7)	0.4279 (5)	0.0808 (16)	
H34A	0.1537	0.9354	0.4181	0.121*	
H34B	0.1565	1.0749	0.3591	0.121*	
H34C	0.1526	1.0593	0.5028	0.121*	
C43	0.36703 (17)	0.9114 (3)	0.3314 (3)	0.0377 (6)	
N41	0.40907 (14)	0.8932 (2)	0.2387 (2)	0.0380 (5)	
C41	0.47758 (17)	0.8488 (3)	0.2747 (3)	0.0396 (6)	
C42	0.4862 (2)	0.8312 (3)	0.3948 (3)	0.0457 (7)	
H42	0.5295	0.8002	0.4325	0.055*	
S42	0.40690 (5)	0.87335 (9)	0.46931 (7)	0.0496 (3)	
C44	0.53543 (17)	0.8225 (3)	0.1831 (3)	0.0406 (7)	
O44	0.59620 (14)	0.7815 (2)	0.2133 (2)	0.0506 (6)	

### Atomic displacement parameters (Å^2^)

**Table:**

	*U*^11^	*U*^22^	*U*^33^	*U*^12^	*U*^13^	*U*^23^
C1	0.114 (7)	0.077 (6)	0.133 (10)	−0.045 (5)	0.000	0.000
C2	0.148 (11)	0.073 (6)	0.168 (13)	0.009 (6)	0.000	0.000
N1	0.116 (6)	0.098 (5)	0.106 (6)	−0.056 (5)	0.000	0.000
N11	0.0380 (13)	0.0362 (13)	0.0512 (14)	−0.0033 (10)	0.0115 (11)	−0.0055 (11)
C11	0.052 (15)	0.038 (10)	0.044 (7)	−0.010 (8)	0.007 (7)	−0.014 (7)
C11'	0.033 (5)	0.044 (11)	0.056 (13)	−0.003 (6)	0.010 (7)	−0.010 (7)
C12'	0.038 (3)	0.066 (5)	0.058 (4)	0.000 (3)	0.001 (3)	−0.015 (4)
C13'	0.061 (4)	0.108 (7)	0.042 (4)	0.002 (5)	0.002 (3)	−0.020 (4)
C14'	0.072 (6)	0.079 (6)	0.092 (8)	−0.014 (5)	0.003 (5)	−0.019 (6)
C15'	0.098 (9)	0.125 (11)	0.125 (10)	−0.022 (9)	−0.025 (8)	−0.055 (10)
C16	0.0473 (17)	0.0529 (18)	0.0400 (15)	−0.0156 (15)	0.0093 (14)	−0.0128 (14)
O16	0.0527 (13)	0.0491 (13)	0.0558 (14)	−0.0133 (11)	0.0054 (11)	0.0011 (11)
N21	0.0476 (15)	0.0455 (15)	0.0582 (16)	−0.0079 (12)	0.0158 (13)	−0.0106 (14)
C21	0.0416 (16)	0.0538 (18)	0.0493 (19)	−0.0067 (14)	0.0093 (13)	−0.0060 (15)
C22	0.0501 (18)	0.074 (2)	0.0449 (18)	−0.0171 (19)	0.0121 (15)	−0.0031 (17)
O22	0.0568 (15)	0.0703 (18)	0.0714 (18)	−0.0174 (14)	0.0051 (13)	0.0133 (15)
C23	0.084 (3)	0.112 (4)	0.048 (2)	−0.028 (3)	0.016 (2)	−0.011 (3)
C24	0.0427 (16)	0.0491 (17)	0.0461 (17)	−0.0031 (14)	0.0028 (13)	0.0028 (14)
O24	0.0681 (16)	0.0656 (17)	0.0508 (13)	−0.0296 (14)	0.0026 (13)	−0.0035 (13)
N31	0.0395 (12)	0.0372 (12)	0.0444 (13)	−0.0020 (11)	−0.0003 (11)	−0.0003 (10)
C31	0.0369 (14)	0.0423 (15)	0.0378 (14)	−0.0009 (12)	0.0027 (11)	−0.0025 (12)
C32	0.0476 (17)	0.061 (2)	0.0428 (16)	0.0097 (16)	0.0065 (14)	−0.0050 (15)
C33	0.081 (3)	0.063 (2)	0.062 (2)	0.006 (2)	0.014 (2)	−0.026 (2)
C34	0.053 (2)	0.118 (4)	0.071 (3)	0.021 (3)	0.009 (2)	−0.022 (3)
C43	0.0417 (15)	0.0330 (13)	0.0383 (14)	0.0008 (11)	0.0043 (12)	0.0015 (11)
N41	0.0410 (12)	0.0297 (10)	0.0432 (12)	0.0017 (10)	0.0068 (11)	0.0010 (10)
C41	0.0384 (14)	0.0285 (12)	0.0518 (17)	0.0003 (11)	0.0065 (13)	0.0025 (12)
C42	0.0450 (17)	0.0422 (16)	0.0499 (18)	0.0029 (14)	0.0035 (14)	0.0072 (14)
S42	0.0482 (4)	0.0615 (5)	0.0391 (4)	0.0057 (4)	0.0043 (3)	0.0082 (4)
C44	0.0408 (15)	0.0242 (11)	0.0568 (18)	−0.0015 (11)	0.0103 (14)	−0.0020 (12)
O44	0.0434 (12)	0.0416 (11)	0.0669 (16)	0.0063 (10)	0.0086 (12)	0.0024 (11)

### Geometric parameters (Å, °)

**Table:**

C1—N1	1.193 (15)	C21—H21A	1.0000
C1—C2	1.305 (19)	C22—O22	1.417 (6)
C2—H2A	0.9800	C22—C23	1.528 (6)
C2—H2B	0.9800	C22—H22A	1.0000
C2—H2C	0.9800	O22—H22	0.84
N11—C44	1.324 (5)	C23—H23A	0.9800
N11—C11'	1.49 (4)	C23—H23B	0.9800
N11—C11	1.49 (5)	C23—H23C	0.9800
N11—H11	0.8800	C24—O24	1.236 (5)
C11—C16	1.51 (5)	C24—N31^i^	1.344 (4)
C11—H11A	0.9900	N31—C24^i^	1.344 (4)
C11—H11B	0.9900	N31—C31	1.451 (4)
C11'—C16	1.51 (5)	N31—H31	0.8800
C11'—C12'	1.55 (4)	C31—C43	1.521 (4)
C11'—H11C	1.0000	C31—C32	1.550 (4)
C12'—C13'	1.534 (11)	C31—H31A	1.0000
C12'—C14'	1.565 (13)	C32—C34	1.529 (6)
C12'—H12	1.0000	C32—C33	1.531 (6)
C13'—H13A	0.9800	C32—H32	1.0000
C13'—H13B	0.9800	C33—H33A	0.9800
C13'—H13C	0.9800	C33—H33B	0.9800
C14'—C15'	1.527 (18)	C33—H33C	0.9800
C14'—H14A	0.9900	C34—H34A	0.9800
C14'—H14B	0.9900	C34—H34B	0.9800
C15'—H15A	0.9800	C34—H34C	0.9800
C15'—H15B	0.9800	C43—N41	1.294 (4)
C15'—H15C	0.9800	C43—S42	1.736 (3)
C16—O16	1.245 (5)	N41—C41	1.389 (4)
C16—N21	1.336 (5)	C41—C42	1.351 (5)
N21—C21	1.469 (4)	C41—C44	1.487 (4)
N21—H21	0.8800	C42—S42	1.719 (4)
C21—C24	1.535 (5)	C42—H42	0.9500
C21—C22	1.538 (5)	C44—O44	1.233 (4)
			
N1—C1—C2	180.000 (3)	C24—C21—H21A	105.8
C1—C2—H2A	109.5	C22—C21—H21A	105.8
C1—C2—H2B	109.5	O22—C22—C23	111.4 (4)
H2A—C2—H2B	109.5	O22—C22—C21	113.5 (3)
C1—C2—H2C	109.5	C23—C22—C21	111.2 (4)
H2A—C2—H2C	109.5	O22—C22—H22A	106.7
H2B—C2—H2C	109.5	C23—C22—H22A	106.7
C44—N11—C11'	126.0 (16)	C21—C22—H22A	106.7
C44—N11—C11	116.1 (17)	C22—O22—H22	109
C44—N11—H11	122.0	C22—C23—H23A	109.5
C11'—N11—H11	111.9	C22—C23—H23B	109.5
C11—N11—H11	122.0	H23A—C23—H23B	109.5
N11—C11—C16	109 (3)	C22—C23—H23C	109.5
N11—C11—H11A	109.8	H23A—C23—H23C	109.5
C16—C11—H11A	109.8	H23B—C23—H23C	109.5
N11—C11—H11B	109.8	O24—C24—N31^i^	122.6 (3)
C16—C11—H11B	109.8	O24—C24—C21	119.7 (3)
H11A—C11—H11B	108.3	N31^i^—C24—C21	117.6 (3)
N11—C11'—C16	109 (3)	C24^i^—N31—C31	121.7 (3)
N11—C11'—C12'	112 (3)	C24^i^—N31—H31	119.1
C16—C11'—C12'	115 (3)	C31—N31—H31	119.1
N11—C11'—H11C	106.7	N31—C31—C43	108.7 (2)
C16—C11'—H11C	106.7	N31—C31—C32	113.6 (3)
C12'—C11'—H11C	106.7	C43—C31—C32	114.4 (3)
C13'—C12'—C11'	110.0 (18)	N31—C31—H31A	106.5
C13'—C12'—C14'	114.1 (8)	C43—C31—H31A	106.5
C11'—C12'—C14'	112.2 (19)	C32—C31—H31A	106.5
C13'—C12'—H12	106.7	C34—C32—C33	111.3 (4)
C11'—C12'—H12	106.7	C34—C32—C31	109.6 (3)
C14'—C12'—H12	106.7	C33—C32—C31	111.9 (3)
C12'—C13'—H13A	109.5	C34—C32—H32	108.0
C12'—C13'—H13B	109.5	C33—C32—H32	108.0
H13A—C13'—H13B	109.5	C31—C32—H32	108.0
C12'—C13'—H13C	109.5	C32—C33—H33A	109.5
H13A—C13'—H13C	109.5	C32—C33—H33B	109.5
H13B—C13'—H13C	109.5	H33A—C33—H33B	109.5
C15'—C14'—C12'	102.8 (9)	C32—C33—H33C	109.5
C15'—C14'—H14A	111.2	H33A—C33—H33C	109.5
C12'—C14'—H14A	111.2	H33B—C33—H33C	109.5
C15'—C14'—H14B	111.2	C32—C34—H34A	109.5
C12'—C14'—H14B	111.2	C32—C34—H34B	109.5
H14A—C14'—H14B	109.1	H34A—C34—H34B	109.5
C14'—C15'—H15A	109.5	C32—C34—H34C	109.5
C14'—C15'—H15B	109.5	H34A—C34—H34C	109.5
H15A—C15'—H15B	109.5	H34B—C34—H34C	109.5
C14'—C15'—H15C	109.5	N41—C43—C31	123.1 (3)
H15A—C15'—H15C	109.5	N41—C43—S42	114.6 (2)
H15B—C15'—H15C	109.5	C31—C43—S42	122.1 (2)
O16—C16—N21	123.0 (3)	C43—N41—C41	110.6 (3)
O16—C16—C11	124.9 (17)	C42—C41—N41	115.6 (3)
N21—C16—C11	112.1 (17)	C42—C41—C44	124.3 (3)
O16—C16—C11'	115.9 (18)	N41—C41—C44	120.1 (3)
N21—C16—C11'	121.0 (18)	C41—C42—S42	109.9 (3)
C16—N21—C21	126.2 (3)	C41—C42—H42	125.1
C16—N21—H21	116.9	S42—C42—H42	125.1
C21—N21—H21	116.9	C42—S42—C43	89.31 (16)
N21—C21—C24	113.0 (3)	O44—C44—N11	123.6 (3)
N21—C21—C22	115.1 (3)	O44—C44—C41	121.0 (3)
C24—C21—C22	110.5 (3)	N11—C44—C41	115.4 (3)
N21—C21—H21A	105.8		
			
C44—N11—C11—C16	−79 (3)	C22—C21—C24—O24	−31.0 (5)
C11'—N11—C11—C16	83 (20)	N21—C21—C24—N31^i^	21.2 (5)
C44—N11—C11'—C16	−62 (3)	C22—C21—C24—N31^i^	151.9 (3)
C11—N11—C11'—C16	−82 (19)	C24^i^—N31—C31—C43	117.0 (3)
C44—N11—C11'—C12'	169.4 (14)	C24^i^—N31—C31—C32	−114.4 (4)
C11—N11—C11'—C12'	149 (22)	N31—C31—C32—C34	70.0 (5)
N11—C11'—C12'—C13'	172 (2)	C43—C31—C32—C34	−164.4 (4)
C16—C11'—C12'—C13'	47 (3)	N31—C31—C32—C33	−54.0 (4)
N11—C11'—C12'—C14'	−60 (3)	C43—C31—C32—C33	71.6 (4)
C16—C11'—C12'—C14'	175 (2)	N31—C31—C43—N41	−27.9 (4)
C13'—C12'—C14'—C15'	21.2 (13)	C32—C31—C43—N41	−156.0 (3)
C11'—C12'—C14'—C15'	−105 (2)	N31—C31—C43—S42	157.8 (2)
N11—C11—C16—O16	−52 (3)	C32—C31—C43—S42	29.6 (4)
N11—C11—C16—N21	126 (2)	C31—C43—N41—C41	−175.8 (3)
N11—C11—C16—C11'	−83 (19)	S42—C43—N41—C41	−1.0 (3)
N11—C11'—C16—O16	−69 (3)	C43—N41—C41—C42	1.3 (4)
C12'—C11'—C16—O16	57 (3)	C43—N41—C41—C44	−179.3 (3)
N11—C11'—C16—N21	114 (2)	N41—C41—C42—S42	−0.9 (4)
C12'—C11'—C16—N21	−119 (2)	C44—C41—C42—S42	179.7 (2)
N11—C11'—C16—C11	82 (18)	C41—C42—S42—C43	0.3 (3)
C12'—C11'—C16—C11	−151 (21)	N41—C43—S42—C42	0.4 (3)
O16—C16—N21—C21	7.0 (6)	C31—C43—S42—C42	175.2 (3)
C11—C16—N21—C21	−171.0 (18)	C11'—N11—C44—O44	−1(2)
C11'—C16—N21—C21	−176.9 (19)	C11—N11—C44—O44	3(2)
C16—N21—C21—C24	65.8 (5)	C11'—N11—C44—C41	179 (2)
C16—N21—C21—C22	−62.5 (5)	C11—N11—C44—C41	−177 (2)
N21—C21—C22—O22	76.3 (4)	C42—C41—C44—O44	0.3 (5)
C24—C21—C22—O22	−53.2 (4)	N41—C41—C44—O44	−179.1 (3)
N21—C21—C22—C23	−50.3 (5)	C42—C41—C44—N11	179.5 (3)
C24—C21—C22—C23	−179.8 (4)	N41—C41—C44—N11	0.1 (4)
N21—C21—C24—O24	−161.7 (4)		

Symmetry codes: (i) −*x*+1, −*y*+2, *z*.

### Hydrogen-bond geometry (Å, °)

**Table:**

*D*—H···*A*	*D*—H	H···*A*	*D*···*A*	*D*—H···*A*
O22—H22···O16	0.84	1.87	2.674 (4)	160
N11—H11···O16^i^	0.88	2.12	2.984 (3)	166
N21—H21···O22^ii^	0.88	2.29	2.953 (4)	132
N21—H21···O24^ii^	0.88	2.25	3.013 (4)	145
N31—H31···O44^i^	0.88	2.27	3.074 (3)	152

Symmetry codes: (i) −*x*+1, −*y*+2, *z*; (ii) −*x*+3/2, *y*−1/2, −*z*.
